# COVID-19 Pandemic School Disruptions and Acute Mental Health in Children and Adolescents

**DOI:** 10.1001/jamanetworkopen.2024.25829

**Published:** 2024-08-05

**Authors:** Chiara Davico, Daniele Marcotulli, Giuseppe Abbracciavento, Thomas Anfosso, Massimo Apicella, Roberto Averna, Marzia Bazzoni, Dario Calderoni, Luca Cammisa, Alessandra Carta, Sara Carucci, Giorgio Cozzi, Federica Di Santo, Elisa Fazzi, Caterina Lux, Chiara Narducci, Lino Nobili, Ilaria Onida, Tiziana Pisano, Umberto Raucci, Idanna Sforzi, Laura Siri, Stefano Sotgiu, Simone Tavano, Arianna Terrinoni, Sara Uccella, Stefano Vicari, Caterina Zanus, Benedetto Vitiello

**Affiliations:** 1 Department of Public Health and Pediatric Sciences, University of Turin/Regina Margherita Children’s Hospital, Turin, Italy; 2 University of Trieste, B. Garofalo Hospital, Trieste, Italy; 3 Department of Clinical and Experimental Science, University of Brescia, Brescia, Italy; 4 Division of Child and Adolescent Psychiatry, Bambino Gesù Children’s Hospital IRCCS, Rome, Italy; 5 Department of Biomedical Sciences, Section of Neuroscience & Clinical Pharmacology, University of Cagliari, Cagliari, Italy; 6 Department of Human Neurosciences, Sapienza University of Rome, Rome, Italy; 7 University Hospital of Sassari, Division of Child Neuropsychiatry, Sassari, Italy; 8 Child & Adolescent Neuropsychiatry Unit, “A. Cao” Paediatric Hospital, ASL Cagliari, Italy; 9 IRCCS Istituto G. Gaslini, University of Genoa, Genoa, Italy; 10 IRCCS Istituto G. Gaslini, Genoa, Italy; 11 Neuroscience Department, Meyer Children’s Hospital IRCCS, Florence, Italy; 12 Emergency Department and Trauma Center Meyer Children’s Hospital IRCCS, Florence, Italy; 13 Dipartimento di Scienze della Vita e Sanità Pubblica, Catholic University, Rome, Italy

## Abstract

**Importance:**

There are suggestions that school pressure may be stressful and a factor in child and adolescent mental health disturbances, but data about this association are scarce and inconclusive.

**Objective:**

To assess whether varying degrees of school interruption were associated with changes in emergency department (ED) psychiatric visits of children and adolescents before and after the COVID-19 outbreak.

**Design, Setting, and Participants:**

A cross-sectional observational study was conducted at 9 urban university hospitals in Italy. All ED visits from January 1, 2018, to December 31, 2021, for psychiatric reasons of patients younger than 18 years were examined for demographic characteristics and type of psychopathologic factors. Data analysis was conducted from July 1 to August 31, 2023.

**Exposure:**

The disruption in the usual succession of school and holiday periods brought on by the COVID-19 pandemic at different times and with various degrees of intensity.

**Main Outcomes and Measures:**

Total number of pediatric ED visits, psychiatric ED visits, and psychiatric ED visits categorized by specific reasons (eg, psychomotor agitation, suicide ideation [SI] or suicide attempt [SA], and eating disorders) on a weekly basis.

**Results:**

A total of 13 014 psychiatric ED visits (1.3% of all pediatric ED visits) were recorded (63.2% females; mean [SD] age, 13.8 [3.8] years). The number of ED psychiatric visits increased over time (incidence rate ratio [IRR], 1.19; 95% CI, 1.16-1.22 for each year). Significant increases in ED visits were observed for eating disorders (294.8%), SI (297.8%), and SA (249.1%). School opening, but not social lockdown restriction, was associated with an increase in the number of ED psychiatric visits (IRR, 1.29; 95% CI, 1.23-1.34), which was evident for females and for SI with SA. Socioeconomic status was associated with an increase in psychiatric visits for males (IRR, 1.12; 95% CI, 1.04-1.20) but not females (IRR, 1.04; 95% CI, 0.98-1.10).

**Conclusions and Relevance:**

In this study, school opening was associated with an increased incidence of acute psychiatric emergencies among children and adolescents, suggesting that school can be a substantial source of stress with acute mental health implications.

## Key Points

**Question:**

Were COVID-19 pandemic–related school disruptions associated with youth mental health emergencies?

**Findings:**

In this cross-sectional study of 13 014 pediatric emergency department visits at 9 university hospitals in Italy, school opening following the disruption was associated with an increase in acute psychiatric emergencies. In particular, visits for suicide ideation/suicide attempt increased during the school calendar and especially among females.

**Meaning:**

The findings of this study suggest that school may be a major source of stress for youths; factors mediating school-associated mental health disturbances in youth should be investigated.

## Teaser

This cross-sectional study examines the number of emergency department visits for acute psychiatric events in children and adolescents in Italy associated with school closures and reopenings during the COVID-19 pandemic.

## Introduction

Concern has been raised about a possible role of school pressure in mental health disturbances in youth, but few studies have investigated whether there is an association.^1^ Examining psychiatric emergency department (ED) visits, which represent the most severe mental disorders, may help evaluate a possible association with school opening. An association between school days and higher rates of ED visits has been found in local samples from the US and UK,^2,3^ especially regarding visits for self-harm and aggression.^4,5^ Investigating possible factors in youth psychiatric emergencies is timely given the steep increase in their number and severity^6,7,8^ and the trend of increasing suicide rates in youth over the past decade.^9,10,11^

The COVID-19 pandemic disrupted school for millions of students worldwide at different times and with variable intensity. This unique situation can offer an opportunity to study the association between school and psychiatric emergencies. The initial wave of the pandemic in the spring of 2020 was accompanied by a marked overall decrease in all pediatric ED visits but a less pronounced decrease for psychiatric visits. The result was a relative increase in the psychiatric/total ED ratio,^12,13^ which was most evident for emergencies due to suicide ideation (SI) with suicide attempt (SA) and those involving females.^8,14,15,16^

A decrease in the rate of adolescent suicides was observed in the US at the onset of the COVID-19 pandemic when schools were closed, with a subsequent increase in the fall of 2020, when many schools returned to in-person instruction.^5,17^ Transitioning from online to in-person schooling was accompanied by a 12% to 18% increase in adolescent suicide.^5,18^ Conversely, there are also suggestions that the prolonged duration of COVID-19 school closure was associated with worse adolescent mental health.^19^

In Italy, the strictest COVID-19 preventive restrictions (social lockdown with limitation of in-person schooling) started in the spring of 2020 and were in force for most of that year, with gradual attenuation, based on the fluctuating incidence of the infection, in 2021. School was online in many areas, at least during some weeks, until the summer of 2021. In-person school reopened in September 2021.^20^

The role of school is confounded by other factors, especially the restrictions to social and recreational activities, so it can be difficult to separate school from social lockdown effects.^21^ The seasonal pattern of ED psychiatric visits should also be considered given higher rates of psychiatric hospitalizations and suicide in spring and summer.^22,23^ Another factor to consider is a general increase in youth psychiatric ED visits worldwide.^6^ Over the past 10 years, the proportion of pediatric ED visits for mental health reasons has approximately doubled, with a 5-fold increase in suicide-related visits.^7,15^

This study used the diverse levels of strictness in the lockdown measures mandated by the health authorities in Italy during the COVID-19 pandemic, which entailed varying degrees of restriction in social contacts, recreational activities, and school opening, including complete school closure, online education, and regular in-person schooling. The level of school restrictions and that of the other social and recreational activities did not always change in parallel, and this discordance allows the association with school to be estimated in the context of the other pandemic measures. Thus, the study aimed to examine whether and how child and adolescent psychiatric ED visits changed in volume, demographic factors, and psychiatric presentations during and soon after the COVID-19 pandemic, with respect to school opening (complete closure, online schooling, and in-person attendance). These analyses accounted for the social containment measures enacted during the pandemic, time trends, and patient’s age, sex, and socioeconomic status.

## Methods

### Design

This was an observational cross-sectional study of the hospital clinical records of all the ED visits of children and adolescents (age, 0-17 years) between January 1, 2018, and December 31, 2021, at 9 university hospitals in Italy. The hospital EDs were located at Brescia, Cagliari, Florence, Genoa, Rome (2 hospitals), Sassari, Trieste, and Turin, and served a cumulative area of about 7 million people. The study consisted of the collection and analysis of anonymized data of naturalistically treated patients, without active recruitment of participants. The study received institutional ethical approval by the ethics committee at Regina Margherita Children's Hospital, Turin. The report followed the Strengthening the Reporting of Observational Studies in Epidemiology (STROBE) reporting guideline.^24^

The hospital medical records were systematically examined by trained clinicians under the supervision of senior child and adolescent psychiatrists (C.D., G.A., T.A., R.A., D.C., A.C., S.C., T.P., A.T., and S.U.) and psychiatric reasons for the ED visit were extracted. Rater reliability was assessed by randomly selecting 5 visits from each participating site and asking 2 raters to categorize them independently. The interrater reliability was 0.90 (Fleiss κ). A complete description of data extraction methods can be found in the eMethods in Supplement 1.

### Variables

School opening was categorized as an ordinal variable from 1 to 3, with in-person schooling assigned the highest value (3), online schooling a medium value (2), and school closure the lowest value (1). For each week since March 2020, lockdown intensity was recorded according to the official administrative categorization for each geographic area. The lockdown severity was quantified using scores from 1 to 5 (the strictest lockdown measures) (eMethods in Supplement 1). The socioeconomic status of each patient’s neighborhood was estimated through the computation of a deprivation index (eMethods in Supplement 1).^25^

### Statistical Analysis

Data analysis was conducted from July 1 to August 31, 2023. Descriptive statistics were applied to the sociodemographic and clinical data. The weekly psychiatric ED visits total count and the weekly psychiatric ED visits count separately for suicide attempts, suicidal ideation, eating disorders, and psychomotor agitation were modeled using generalized mixed models, with visit count following a Poisson distribution in a bayesian framework. Each model had fixed effects for year, school opening, lockdown severity, and median deprivation index. All regression models included study sites as a random intercept, and we allowed the regression slopes for time (both yearly and weekly variations) to differ across study sites. For models assessing SI with SA, we considered the weekly sum of the visits for SI with SA as the dependent variable. eMethods in Supplement 1 provides more detail.

## Results

A total of 1 017 885 pediatric ED visits occurred during the study period: 13 014 (1.3%) were psychiatric visits. Mean (SD) age was 13.8 (3.8) years, 63.2% of the individuals were females, and 36.8% were males.

The total number of pediatric ED visits was significantly reduced in 2020 and 2021 compared with 2018 and 2019 (eTable 1 in Supplement 1). The psychiatric visit numbers were 2655 in 2018 (0.9%), 3136 in 2019 (1.0%), 2563 in 2020 (1.4%), and 4660 in 2021 (2.0%). The proportion of psychiatric visits for females was 61.4% in 2018, 58.2% in 2019, 60.5% in 2020, and 69.1% in 2021. In 2018, the mean (SD) age at presentation (13.47 [4.2] years) was significantly lower (analysis of variance with post hoc Tukey-corrected comparisons, *F*_3,1310_ = 9.70; *P* < .001) than in the subsequent years (13.91 [3.5] years for 2019; 13.75 [4.0] years for 2020; and 13.94 [3.8] years for 2021) (Table 1). A large heterogeneity in the number of psychiatric visits was found among the 9 study sites, which is described in eTable 1 in Supplement 1.

**Table 1.** Demographic and Clinical Characteristics of Patients With ED Psychiatric Visits

Characteristic	No. (%)				
	2018 (n = 2655)	2019 (n = 3136)	2020 (n = 2563)	2021 (n = 4660)	Total (N = 13 014)
Sex					
Male	1026 (38.6)	1312 (41.8)	1013 (39.5)	1441 (30.9)	4792 (36.8)
Female	1629 (61.4)	1824 (58.2)	1550 (60.5)	3219 (69.1)	8222 (63.2)
Mean (SD) age, y	13.47 (4.2)	13.91 (3.5)	13.75 (4.0)	13.94 (3.8)	13.80 (3.8)
ED visit reason					
Psychomotor agitation	1011 (38.1)	1092 (34.8)	850 (33.2)	1354 (29.1)	4307 (33.1)
Anxiety	471 (17.7)	632 (20.2)	369 (14.4)	623 (13.4)	2095 (16.1)
Mood disorder	109 (4.1)	101 (3.2)	98 (3.8)	213 (4.6)	521 (4.0)
Eating disorder	214 (8.1)	245 (7.8)	270 (10.5)	631 (13.5)	1360 (10.4)
Somatic symptoms	151 (5.7)	237 (7.6)	201 (7.8)	255 (5.5)	844 (6.5)
Psychosis	74 (2.8)	121 (3.9)	89 (3.5)	92 (2.0)	376 (2.9)
Self-harm	120 (4.5)	145 (4.6)	119 (4.6)	309 (6.6)	693 (5.3)
Suicidal ideation	189 (7.1)	167 (5.3)	222 (8.7)	563 (12.1)	1141 (8.8)
Suicidal attempt	187 (7.0)	226 (7.2)	238 (9.3)	466 (10.0)	1117 (8.6)
Drug abuse	23 (0.9)	19 (0.6)	22 (0.8)	55 (1.2)	119 (0.9)
Maltreatment	47 (1.8)	56 (1.8)	46 (1.8)	45 (1.0)	194 (1.5)
Tics	48 (1.8)	75 (2.4)	36 (1.4)	35 (0.7)	194 (1.5)
Other	11 (0.4)	20 (0.6)	3 (0.1)	19 (0.4)	53 (0.4)

Abbreviation: ED, emergency department.

The main reasons for the psychiatric ED visits were psychomotor agitation (33.1%), anxiety (16.1%), eating disorders (10.4%), suicidal ideation (8.8%), and suicide attempts (8.6%) (Table 1). These last 3 factors significantly increased during the study period, with increments of 294.8% for eating disorders, 297.8% for SI, and 249.1% for SA.

Tables 2, 3, and 4 present the incidence rate ratios (IRRs) for the variables under study for all psychiatric visits, psychomotor agitation, eating disorders, and SI with SA. eTables 2-7 in Supplement 1 detail the IRRs for psychiatric visits by sex, age (<14 years vs ≥14 years), and psychiatric visits due to suicide attempt.

**Table 2.** Longitudinal Mixed Model With Poisson Distribution of All Psychiatric ED Visits

Variable	IRR (95% CI)	
	All psychiatric visits, No.	All psychiatric visits over total pediatric ED visits, No.
Year	1.19 (1.16-1.22)	1.25 (1.22-1.28)
School	1.29 (1.23-1.34)	1.12 (1.08-1.17)
Lockdown severity	0.77 (0.71-0.83)	1.18 (1.09-1.28)
Deprivation index	1.02 (0.97-1.07)	1.02 (0.97-1.07)

Abbreviations: ED, emergency department; IRR, incidence rate ratio.

**Table 3.** Longitudinal Mixed Model With Poisson Distribution of ED Visits for Eating Disorders

Variable	IRR (95% CI)	
	Eating disorders visits, No.	Eating disorders visits over total pediatric ED visits offset, No.
Year	1.41 (1.30-1.53)	1.48 (1.36-1.61)
School	1.22 (1.07-1.41)	1.07 (0.94-1.22)
Lockdown severity	0.58 (0.44-0.76)	0.88 (0.66-1.15)
Deprivation index	0.92 (0.75-1.12)	0.95 (0.78-1.15)

Abbreviations: ED, emergency department; IRR, incidence rate ratio.

**Table 4.** Longitudinal Mixed Model With Poisson Distribution of ED Visits for SI With SA

Variable	IRR (95% CI)	
	All SI with SA visits, No.	All SI with SA visits over total ED pediatric visits, No.
Year	1.38 (1.30-1.47)	1.46 (1.03-3.39)
School	1.32 (1.20-1.47)	1.18 (1.04-1.54)
Lockdown severity	0.78 (0.66-0.94)	1.18 (0.34-3.47)
Deprivation index	0.99 (0.86-1.12)	0.57 (0.20-1.10)

Abbreviations: ED, emergency department; IRR, incidence rate ratio; SA, suicide attempt; SI, suicidal ideation.

### Overall Trends of Psychiatric Visits

Monthly trends of psychiatric ED visits are shown in the Figure. To explore the main factors associated with the weekly trends of psychiatric visits, we used a longitudinal mixed model applied to a Poisson distribution. School opening was associated with the largest increment in the number of visits (IRR, 1.29; 95% CI, 1.23-1.34) (Table 2, Figure). A 19% increase in psychiatric visits was also found for every year since the beginning of the study (IRR, 1.19; 95% CI, 1.16-1.22) (Table 2, Figure). Additionally, lockdown severity was negatively associated with the weekly number of psychiatric visits (IRR, 0.77; 95% CI, 0.71-0.83) (Table 2, Figure). No association was found for the deprivation index (IRR, 1.02; 95% CI, 0.97-1.07) (Table 2).

**Figure.**
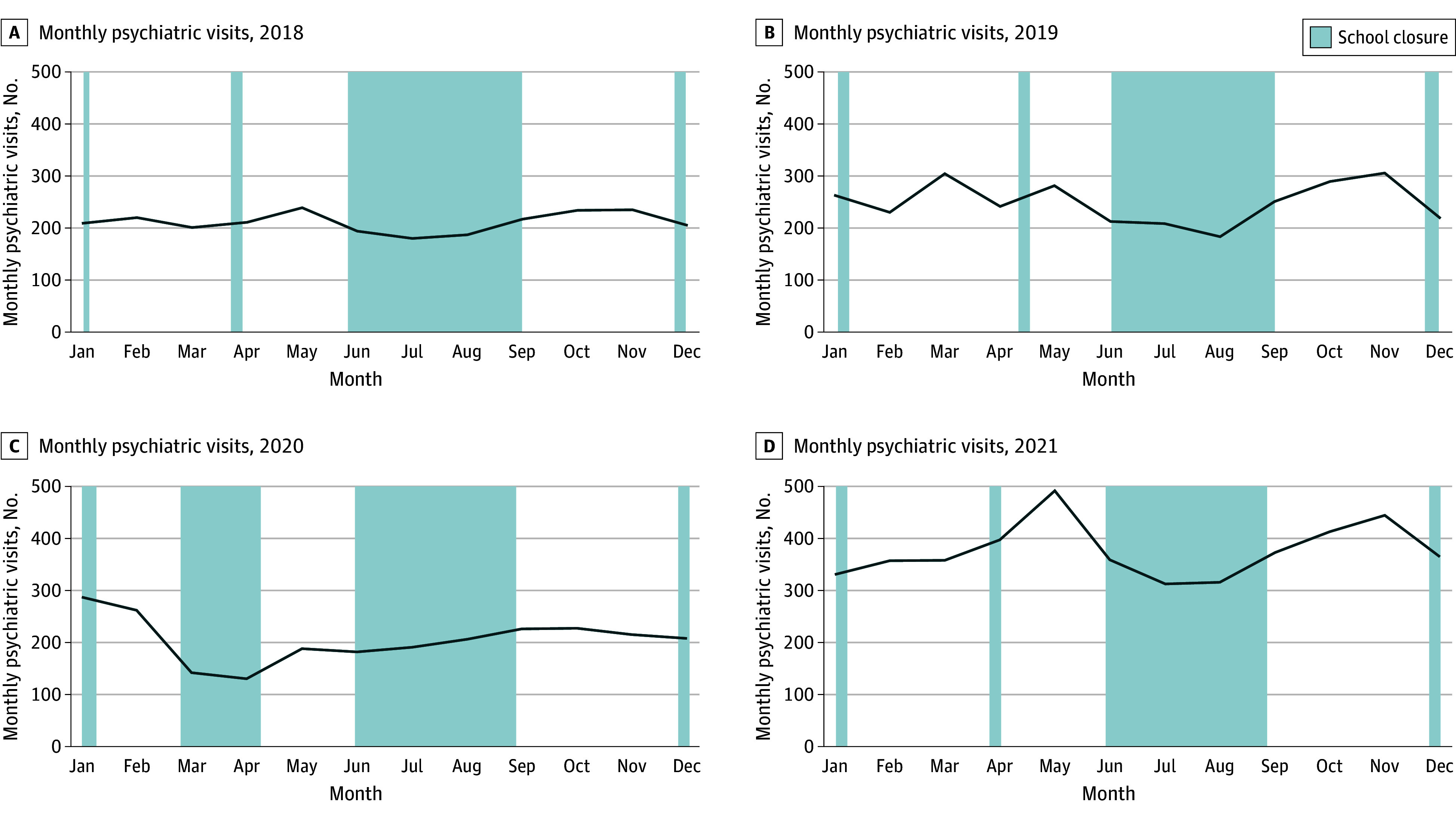
Monthly Psychiatric Visits, Years 2018-2021 Monthly trends of psychiatric visits per year.

To examine whether the observed findings were specific to psychiatric visits or mirroring other pediatric conditions, we incorporated an offset for the weekly number of all the pediatric visits into the same model. The analysis revealed that, when accounting for the total number of pediatric ED visits, the lockdown severity was associated with an increase of the proportion of psychiatric visits over the total (IRR, 1.18; 95% CI, 1.09-1.28) (Table 2), while the associations observed for the other analyzed variables were similar to the model without the offset (Table 2).

Given this difference between the 2 models (with and without the offset) and the need for exploring the specificity of mental health conditions while accounting for the changing trends of ED visits, we decided to incorporate, for all the subsequent models (Table 3 and Table 4; eTables 2-7 in Supplement 1), an offset for the weekly number of pediatric visits over the total number of pediatric ED visits. As a sensitivity analysis, we also analyzed the same models without incorporating the offset and found that the main difference was in the lockdown severity index. Specifically, when the offset was not incorporated in the model, the severity index was inversely associated with the number of visits (Table 2, Table 3, and Table 4; eTables 2-7 in Supplement 1). Unless otherwise stated, the models presented in the text include the offset for total ED visits, and the tables present results for models with and without offset (sensitivity analyses).

### The Role of School

School opening was associated with an increase in the weekly proportion of psychiatric visits for females (IRR, 1.16; 95% CI, 1.10-1.22) (eTable 2 in Supplement 1) but not for males (IRR, 1.03; 95% CI, 0.97-1.10) (eTable 3 in Supplement 1). School opening was associated with a higher proportion of psychiatric visits both in younger (IRR, 1.09; 95% CI, 1.02-1.16) (eTable 4 in Supplement 1) and older (IRR, 1.10; 95% CI, 1.05-1.16) patients (eTable 5 in the Supplement 1). The proportion of visits for eating disorders was not associated with in-person schooling (IRR, 1.07; 95% CI, 0.94-1.22) (Table 3). Conversely, the proportion of visits for SI with SA taken together or visits for SA only increased with school opening (for SI with SA, IRR, 1.18; 95% CI, 0.34-3.47 [Table 4] and for SA only, IRR, 1.08; 95% CI, 0.95-1.24 [eTable 6 in Supplement 1]).

### The Role of Lockdown Severity

Lockdown severity was associated with an increase in the proportion of psychiatric visits both for females (IRR, 1.17; 95% CI, 1.05-1.29) (eTable 2 in Supplement 1) and males (IRR, 1.16; 95% CI, 1.02-1.31) (eTable 3 in Supplement 1). When considering all the psychiatric visits, the association with lockdown severity was evident in older adolescents (IRR, 1.22; 95% CI, 1.10-1.35) (eTable 5 in Supplement 1) but not patients younger than 14 years (IRR, 1.07; 95% CI, 0.94-1.22) (eTable 4 in Supplement 1).

No association with lockdown severity was found for ED visits for eating disorders (IRR, 0.88; 95% CI, 0.66-1.15) (Table 3), SI with SA (IRR, 1.18; 95% CI, 0.34-3.47) (Table 4), or SA only (included also in the previous category of SI with SA) (IRR, 1.07; 95% CI, 0.85-1.40) (eTable 6 in Supplement 1). Conversely, the proportion of visits for psychomotor agitation increased (IRR, 1.28; 95% CI, 1.12-1. 46) (eTable 7 in Supplement 1).

### The Role of Year

Regression analysis over the 4-year period (2018-2021) showed a significant temporal trend, with year being positively associated with increased number and proportion of psychiatric visits. For the psychiatric visits overall, there was a notable increase in both females (IRR, 1.29; 95% CI, 1.25-1.33) (eTable 2 in Supplement 1) and males (IRR, 1.18; 95% CI, 1.14-1.23) (eTable 3 in Supplement 1). Similar findings were observed for younger (IRR, 1.20; 95% CI, 1.15-1.25) (eTable 4 in Supplement 1) and older (IRR, 1.29; 95% CI, 1.25-1.33) patients (eTable 5 in Supplement 1). Increase over time was found for eating disorders (IRR, 1.48; 95% CI, 1.36-1.61) (Table 3), SI with SA (IRR, 1.46; 95% CI, 1.03-3.39) (Table 4), SA alone (IRR, 1.43; 95% CI, 1.31-1.56) (eTable 6 in Supplement 1), and psychomotor agitation (IRR, 1.13; 95% CI, 1.08-1.18) (eTable 7 in Supplement 1).

### The Role of Socioeconomic Status

Deprivation index was associated with a higher proportion of psychiatric visits in males (IRR, 1.12; 95% CI, 1.04-1.20) (eTable 3 in Supplement 1) but not females (IRR, 1.04; 95% CI, 0.98-1.10) (eTable 2 in Supplement 1), controlled for age (eTables 4 and 5 in Supplement 1). Deprivation index was not associated with ED visits for eating disorders (IRR, 0.95; 95% CI, 0.78-1.15) (Table 3), SI with SA (IRR, 0.57; 95% CI, 0.20-1.10) (Table 4), or SA alone (IRR, 0.98; 95% CI, 0.80-1.15) (eTable 6 in Supplement 1).

## Discussion

By assessing hospital ED visits before and during the COVID-19 pandemic, we found that school opening was associated with an increase in ED psychiatric visits. The variability in the restrictions imposed by the authorities during the pandemic and school closure during the holidays allowed the school association to be estimated while accounting for other social restrictions, such as sport and recreational activities. In particular, the school association was evident for female adolescents and for ED visits due to SI with SA.

The steep increase in potentially life-threatening conditions, including eating disorders and SI with SA, in this Italian sample is in line with reports from other countries.^6,7,8,14,26^ The study findings are also consistent with a previously reported association between school calendar and ED psychiatric visits and between in-person school and suicide in children and adolescents.^5,18^ Student-reported school climate has been recently identified as a significant factor in mental health among adolescents.^26,27^ Attention has been placed on academic pressure as a possible source of stress and mental health problems.^1^ School represents the main setting for peer interaction and interface over social issues and academic performance. While it constitutes an important incentive and a resource for socialization and self-realization,^28^ school can also be stressful for vulnerable youth.^29^

Other factors may be at play in decreasing psychiatric emergencies when school is not in session, possibly causing a spike on reopening. For some students, the avoidance of stress-provoking social situations in school may translate into a temporary relief when school is closed. The lack of habituation to managing social stress during school closure may then be associated with greater distress upon school reopening. Possible stress from risk of contagion upon returning to school is another factor to consider.

In our study, it was not possible to separate the role of school-associated social stressors (eg, bullying, stress as a minoritized individual, and peer conflict) from that of academic stress (ie, grades, examinations, and evaluations). In searching for possible explanations for the increasing rates of youth psychiatric emergencies, one could consider whether academic pressure has been on the increase over recent years, but there is little evidence for this. Another possible explanation may be found in lower levels of resilience among youth, who may have been less able to cope with multiple stressors, including the COVID-19 pandemic. It is worth considering that the pandemic might have been a factor in the acceleration of use of social media by youth.^30^

In-person schooling was associated with an increase in ED visits for SI with SA but not for eating disorders (Table 2). Usually, SI with SA is an acute emergency requiring immediate attention, while eating disorders develop slowly, thus complicating whether there is an association with the school calendar. Another hypothesis is that more time spent at home may magnify the eating disorder psychopathologic factors.

The association between school opening and increase in ED psychiatric visits was evident for females but not males. The variation by sex raises the issue of possible differences in coping with academic pressure. In a large cross-national study, girls were more likely to perceive schoolwork pressure than boys.^29^ Sex differences were also suggested by a recent report that prolonged school closure in Germany was associated with more marked worsening in mental health among boys.^19^

An unexpected finding was that coming from a lower socioeconomic status neighborhood was associated with an increased rate of psychiatric ED visits only among males (eTable 2 in Supplement 1). Few studies have examined sex as a possible factor in the association between socioeconomic status and child mental health.^31,32^

Greater severity of the pandemic lockdown was associated with both a decreased number of ED visits overall and an increase in the proportion of psychiatry ED visits. From a public health perspective, this finding points to a relatively greater relevance of mental health concerns among children and adolescents during the pandemic and to the need for adequate access to psychiatric services during emergencies. Several factors might have contributed to the general decrease in ED visits during the social lockdown, such as fear of contagion at the hospital, lower risk of trauma and infections, and higher parental supervision. Our study found that social isolation and school closure were not associated with an acute destabilization of mental health. It is possible, however, that the cumulative outcome of prolonged social isolation over time might have contributed to a progressive increase in the number of psychiatric ED visits, as indicated by the observed association with the year. These findings should be considered in the context of the increasing number of psychiatric ED visits of children and adolescents that was evident even before the pandemic.^7,9,10,11^

### Limitations

This study has several limitations. The sample was large but not epidemiologically drawn and may not be representative of the entire population. However, all pediatric ED visits were comprehensively assessed at 9 major university hospitals, whose catchment areas cumulatively account for a large portion of the urban population in Italy. Another limitation is the time frame of only 4 years, which prevents assessing the trend in psychiatric ED visits antecedent to the pandemic. In addition, while we can distinguish the role of lockdown severity from that of school opening, we cannot distinguish between social and recreational activities and school, since both followed a similar time course.

## Conclusions

This cross-sectional study found an association between school opening and increase in acute psychiatric emergencies. The results support the view that school may be a significant source of stress for youth and point to the need to investigate possible contributing factors, such as perceived academic pressure, individual vulnerabilities, parental expectations, and social stress from peer interaction, as potential mediators of school-associated mental health disturbances in youth. Future research should address differences in psychological well-being of students by school system as well as the association between school calendar and psychiatric ED visits.

## Notes

Supplement 1. **eTable 1.** Emergency Department Visits (EDs) by Children (<18 Years) at the Study Sites **eTable 2.** Longitudinal Mixed Model With Poisson Distribution for Females’ Psychiatric Visits **eTable 3.** Longitudinal Mixed Model With Poisson Distribution for Males’ Psychiatric Visits **eTable 4.** Longitudinal Mixed Model With Poisson Distribution for Young Patient’s (<14 Years of Age) Psychiatric Visits **eTable 5.** Longitudinal Mixed Model With Poisson Distribution for Older Patients’ (>14 Years) Psychiatric Visits **eTable 6.** Longitudinal Mixed Model With Poisson Distribution for Psychiatric Visits for Suicide Attempt **eTable 7.** Longitudinal Mixed Model With Poisson Distribution of ED Visits for Psychomotor Agitation **eMethods.** Detailed Methods

Supplement 2. **Nonauthor Collaborators.** The Italian Covid-Child and Adolescent Psychiatric Emergencies Study Group

Supplement 3.
Data Sharing Statement
