# Supplementary material for: COVID-19 Pandemic School Disruptions and Acute Mental Health in Children and Adolescents
Source: JAMA Netw Open. 2024 Aug 5;7(8):e2425829. doi: 10.1001/jamanetworkopen.2024.25829 (PMC11301547; doi:10.1001/jamanetworkopen.2024.25829)
Supplement: Supplement 1. — eTable 1. Emergency Department Visits (EDs) by Children (<18 Years) at the Study Sites eTable 2. Longitudinal Mixed Model With Poisson Distribution for Females’ Psychiatric Visits eTable 3. Longitudinal Mixed Model With Poisson Distribution for Males’ Psychiatric Visits eTable 4. Longitudinal Mixed Model With Poisson Distribution for Young Patient’s (<14 Years of Age) Psychiatric Visits eTable 5. Longitudinal Mixed Model With Poisson Distribution for Older Patients’ (>14 Years) Psychiatric Visits eTable 6. Longitudinal Mixed Model With Poisson Distribution for Psychiatric Visits for Suicide Attempt eTable 7. Longitudinal Mixed Model With Poisson Distribution of ED Visits for Psychomotor Agitation eMethods. Detailed Methods [file jamanetwopen-e2425829-s001.pdf]

Supplementary Online Content

Davico C, Marcotulli D, Abbracciavento G, et al. COVID-19 pandemic school disruptions and acute mental health status among children and adolescents in Italy. *JAMA Netw Open*. 2024;7(8):e2425829. doi:10.1001/jamanetworkopen.2024.25829

eTable 1. Emergency Department Visits (EDs) by Children (<18 Years) at the Study Sites

eTable 2. Longitudinal Mixed Model With Poisson Distribution for Females' Psychiatric Visits

eTable 3. Longitudinal Mixed Model With Poisson Distribution for Males' Psychiatric Visits

eTable 4. Longitudinal Mixed Model With Poisson Distribution for Young Patient's (<14 Years of Age) Psychiatric Visits

eTable 5. Longitudinal Mixed Model With Poisson Distribution for Older Patients' (>14 Years) Psychiatric Visits

eTable 6. Longitudinal Mixed Model With Poisson Distribution for Psychiatric Visits for Suicide Attempt

eTable 7. Longitudinal Mixed Model With Poisson Distribution of ED Visits for Psychomotor Agitation

eMethods. Detailed Methods

This supplementary material has been provided by the authors to give readers additional information about their work.

© 2024 Davico C et al. *JAMA Network Open.*

eTable 1

eTable 2

eTable 3

eTable 4

eTable 5

eTable 6

eTable 7

Supplementary Methods

Data extraction
Lockdown severity
Socioeconomic status
Statistical analysis

© 2024 Davico C et al. *JAMA Network Open.*

eTable 1- Emergency department visits (EDs) by children (<18 years) at the study sites.

	2018			2019			2020			2021
Sites	All	Psy	%[a]	All	Psy	%[a]	All	Psy	%[a]	All
All sites	294572	2655	0.9	309835	3136	1.0	184608	2563	1.4	228870
Cagliari	17323	110	0.6	20114	228	1.1	9447	144	1.5	12051
Brescia	39805	365	0.9	39286	356	0.9	22691	250	1.1	32149
Florence	40315	226	0.6	42773	290	0.7	24636	258	1.0	32443
Genoa	31720	125	0.4	35960	150	0.4	23175	160	0.7	24994
Rome-*Sapienza*	31298	440	1.4	32092	505	1.6	14257	333	2.3	17270
Rome-*Bambino Gesù*	54081	756	1.4	58302	874	1.5	43733	818	1.90	49754
Sassari	12169	151	1.2	12749	205	1.6	5929	101	1.7	7353
Trieste	24599	160	0.7	24769	158	0.6	15516	113	0.7	19824
Turin	43262	322	0.7	43790	370	0.8	25224	386	1.5	33032

[a]Percent proportion of all visits.

© 2024 Davico C et al. *JAMA Network Open.*

eTable 2- Longitudinal mixed model with Poisson distribution for females' psychiatric visits *.

	All females' psychiatric visits (n) at the ED		*All females' psychiatric visits (n) over total pediatric visits at the ED*	
Variables	*IRR*	*95% CI*	*IRR*	*95% CI*
Year	1.23	**1.19 – 1.27**	1.29	**1.25 – 1.33**
School	1.33	**1.26 – 1.4**	1.16	**1.10 – 1.22**
Lockdown Severity	0.76	**0.69 – 0.84**	1.17	**1.05 – 1.29**
Deprivation Index	1.04	0.98 – 1.10	1.04	0.98 – 1.10

*Longitudinal mixed model with Poisson distribution, with the number of indicated psychiatric visits (left), or the ratio between psychiatric and total pediatric visits (right), as the dependent variable, and year, school, lockdown severity and deprivation index as independent variables.

© 2024 Davico C et al. *JAMA Network Open.*

eTable 3- Longitudinal mixed model with Poisson distribution for males' psychiatric visits.

	All males' psychiatric visits at the ED		*All males' psychiatric visits over total pediatric visits at the ED*	
Variables	*IRR*	*95% CI*	*IRR*	*95% CI*
Year	1.12	**1.07 – 1.16**	1.18	**1.14 – 1.23**
School	1.19	**1.12 – 1.27**	1.03	0.97 – 1.10
Lockdown Severity	0.77	**0.68 – 0.87**	1.16	**1.02 – 1.31**
Deprivation Index	1.13	1.05 – 1.21	1.12	**1.04 – 1.20**

*Longitudinal mixed model with Poisson distribution, with the number of indicated psychiatric visits (left), or the ratio between psychiatric and total pediatric visits (right), as the dependent variable, and year, school, lockdown severity and deprivation index as independent variables.

© 2024 Davico C et al. *JAMA Network Open.*

eTable 4 - Longitudinal mixed model with Poisson distribution for young patient’s (<14 years of age) psychiatric visits *.

	All young patients’ (<14 years) psychiatric visits (n) at the ED		*All young patients’ (<14 years) psychiatric visits (n) over total pediatric visits at the ED*	
Variables	*IRR*	*95% CI*	*IRR*	*95% CI*
Year	1.14	**1.09 - 1.18**	1.20	**1.15-1.25**
School	1.25	**1.17 - 1.34**	1.09	**1.02-1.16**
Lockdown Severity	0.73	**0.64 - 0.82**	1.07	0.94-1.22
Deprivation Index	1.11	**1.04 - 1.18**	1.11	**1.04-1.18**

*Longitudinal mixed model with Poisson distribution, with the number of indicated psychiatric visits (left), or the ratio between psychiatric and total pediatric visits (right), as the dependent variable, and year, school, lockdown severity and deprivation index as independent variables

© 2024 Davico C et al. *JAMA Network Open.*

eTable 5 - Longitudinal mixed model with Poisson distribution for older patients' (>14 years) psychiatric visits *.

	All older patients' (>14 years) psychiatric visits (n) at the ED		*All older patients' (≥14 years) psychiatric visits (n) over total pediatric visits at the ED*	
Variables	*IRR*	*95% CI*	*IRR*	*95% CI*
Year	1.23	**1.19 - 1.27**	1.29	**1.25 - 1.33**
School	1.27	**1.20 - 1.34**	1.10	**1.05 - 1.16**
Lockdown Severity	0.80	**0.72 - 0.88**	1.22	**1.10 - 1.35**
Deprivation Index	1.09	**1.03 - 1.16**	1.08	**1.01 - 1.14**

*Longitudinal mixed model with Poisson distribution, with the number of indicated psychiatric visits (left), or the ratio between psychiatric and total pediatric visits (right), as the dependent variable, and year, school, lockdown severity and deprivation index as independent variables.

© 2024 Davico C et al. *JAMA Network Open.*

eTable 6 - Longitudinal mixed model with Poisson distribution for psychiatric visits for suicide attempt*.

	All suicide attempts visits at the ED		*All suicide attempts visits over total pediatric visits at the ED*	
Variables	*IRR*	*95% CI*	*IRR*	*95% CI*
Year	1.35	**1.24 – 1.47**	1.43	**1.31 – 1.56**
School	1.26	**1.09 – 1.46**	1.08	0.95 – 1.24
Lockdown Severity	0.71	**0.54 – 0.92**	1.07	0.85 – 1.40
Deprivation Index	0.95	0.78 – 1.14	0.98	0.80 – 1.15

*Longitudinal mixed model with Poisson distribution, with the number of indicated psychiatric visits (left), or the ratio between psychiatric and total pediatric visits (right), as the dependent variable, and year, school, lockdown severity and deprivation index as independent variables.

© 2024 Davico C et al. *JAMA Network Open.*

eTable 7 - Longitudinal mixed model with Poisson distribution of ED visits for psychomotor agitation *

	Psychomotor agitation visits (n)		*Psychomotor agitation visits (n) over total pediatric ED visits*	
Variables	*IRR*	*95% CI*	*IRR*	*95% CI*
Year	1.07	**1.03 – 1.12**	1.13	**1.08 – 1.18**
School	1.15	**1.07 – 1.24**	1.01	0.94 – 1.08
Lockdown Severity	0.83	**0.72 – 0.94**	1.28	**1.12 – 1.46**
Deprivation Index	1.01	0.92 – 1.11	1.01	0.93 – 1.10

* Longitudinal mixed model with Poisson distribution, with the number of indicated psychiatric visits (left), or the ratio between psychiatric and total pediatric visits (right), as the dependent variable, and year, school, lockdown severity and deprivation index as independent variables.

© 2024 Davico C et al. *JAMA Network Open.*

dMethods. Detailed Methods

Data extraction

The data were manually extracted from the hospital clinical records. All the ED visits that involved a psychiatric evaluation were identified. The primary psychiatric reason for the ED visit was categorized, in decreasing order of severity, into suicide attempt, psychomotor agitation, psychosis, eating disorders, suicidal ideation, non-suicidal self-injury (NSSI), drug abuse, somatic symptoms disorder, mood disorder, anxiety, maltreatment, sleep disorders, or other. When more than one category applied, the most severe category was selected. Reliability between centers was assessed by randomly selecting 5 ED visits from each participating site and asking two raters to categorize them independently. The inter-rater reliability was 0.90 (Fleiss-k). Cases with doubtful assignment were discussed among raters until a consensus was reached. The patient's ZIP code was extracted to identify residence area and the associated socio-economic status (Rosano, 2020).

Lockdown severity

The intensity of the lockdown was centrally mandated by the Italian Government and modified over time based on the course of the pandemic in each administrative region. For each week since March 2020, the intensity of the lockdown was recorded according to the official administrative categorization. The intensity of the lockdown, or lockdown severity (LS), was quantified using a Likert-type scale with scores from LS-1 to LS-5 (the strictest lockdown measures). Specific descriptors of the LS grades were: LS-1: no restrictions in social contacts, work activities or travel, besides the requirement to wear a mask in social contacts; schools are open as usual; LS-2: restaurants are open but not in the evening, recreational activities are not allowed, movements within region are free, movements between regions are not allowed, no limitations to routine health care; high schools students attended classes by distance learning, lower grades schools are open for students up to 14 years old; LS-3: restaurants are closed, recreational activities not allowed, circulation allowed only within your own town, social contact are limited to two people per day, no limitations to routine health care; high schools students attend classes by distance learning, lower grades schools are open for students up to 11 years old; LS-4: travel is not allowed, 24-hour curfew, leaving home is allowed only for essential activities, restaurants and non-essential businesses are closed, recreational activities not allowed, no limitations to routine health care, most schools are closed, with distance learning allowed; LS-5: like previous level, and, in addition, all routine health suspended as medical services were limited to essential needs and urgent care; all schools are closed or use only distance learning. This grading corresponded to the color-coded categories of COVID-19 risk used by the Italian Government since November 2020 for the weekly classification of each administrative region of the country based on the local pandemic conditions: green (LS-1), yellow (LS-2), orange (LS-3), and red (LS-4). In addition, we added a fifth category (LS-5) to designate the strictest lockdown measures that were imposed at the national level during the first wave of the pandemic, starting on February 23, 2020, and until May 16, 2020.

Socio-economic status

SES of each patients' neighborhood was estimated through the computation of a deprivation index (DI) according to the methods by Rosano et al. 2020. Based on the postal code of the patient mail address and the latest census national database, the DI for each census zone was calculated as the sum of standardized indicators of low level of education, being unemployed, living on rent, living in a crowded house, and living in a single-parent family. Patients were then assigned the DI corresponding to their home postal code.

© 2024 Davico C et al. *JAMA Network Open.*

Statistical analyses

Statistical analyses were performed using the statistical programming language R (version 4.1.2; R Core Team, 2020). Descriptive statistics was applied to sociodemographic and clinical data. Continuous variables were described by mean and standard deviation (SD), and categorical data as percentages. z-test for proportion was used to evaluate differences between the proportion of ED psychiatric visits over total ED visits in 2020 vs 2019. Sex differences, as well as the distributions of the reasons for psychiatric visits across the four years considered, were evaluated with the chi square independence test. Proportion differences across the four years for each psychiatric reason were evaluated with a z-test for proportions, and false discovery rate correction was applied for multiple comparisons p-value correction.

The weekly total ED psychiatric visits count, and weekly ED psychiatric visits count for suicide attempts, suicidal ideation, eating disorders, and psychomotor agitation were modeled using generalized linear mixed models with visit count following a Poisson distribution in a Bayesian framework (using the *brms* package, Bürkner 2017). Each model had fixed effects for year, school opening (described above), lockdown severity (described above), and median deprivation index. All regression models included study sites as a random intercept, and we allowed the regression slopes for time (both yearly and weekly variations) to vary as random effects across study sites. This approach was taken to account for potential heterogeneity in temporal trends among the different study sites. Predictors' collinearity and, in particular, lockdown severity and school opening collinearity, was excluded using variance inflation factor (<5) and by inspecting posterior distributions. Zero-inflated models were used as appropriate for analyzing the trends of ED visits for the less frequent reasons with frequent 0s in the smallest study sites. For each model we report the estimates, and 95% credibility interval; posterior predictive values were graphically checked. As a sensitivity analysis, we re-evaluated our models using negative binomial distributions to account for potential overdispersion in the count data. Upon comparison, we observed that the parameter estimates, associated uncertainty (e.g., 95% credibility intervals), and overall conclusions drawn from the models remained consistent with our primary analysis using Poisson distributions, indicating robustness in our results.

For models assessing suicidality we considered the weekly sum of the visits for suicidal attempt and suicidal ideation ("suicidality") as the dependent variable.

© 2024 Davico C et al. *JAMA Network Open.*
