# Supplementary material for: COVID-19 Pandemic School Disruptions and Acute Mental Health in Children and Adolescents
Source: JAMA Netw Open. 2024 Aug 5;7(8):e2425829. doi: 10.1001/jamanetworkopen.2024.25829 (PMC11301547; doi:10.1001/jamanetworkopen.2024.25829)
Supplement: Supplement 2. — Nonauthor Collaborators. The Italian Covid-Child and Adolescent Psychiatric Emergencies Study Group [file jamanetwopen-e2425829-s002.pdf]

Supplemental Online Content: Nonauthor Collaborators
*First name, last name, and suffix (if applicable) are required and will appear in PubMed.

***Group Name(s): The Italian Covid-Child and Adolescent Psychiatric Emergencies Study Group**

*First Name and Middle Initial(s)	*Last Name	*Suffix (eg, Jr, III)	Academic Degrees	Institution	Location (city, state/province, country)	Role or Contribution, eg, chair, principal investigator	Group (if more than 1 Group listed in the byline) and/or Subgroup (eg, Steering Committee)
**University of Turin**							
Chiara	Davico		MD	University of Turin/Regina Margherita C	Turin, Italy	conceptualization, data curation, supervision, project management; writing - original draft	
Daniele	Marcotulli		MD, PhD	University of Turin/Regina Margherita C	Turin, Italy	conceptualization, data curation, formal analysis, supervision, writing - original draft	
Caterina M.	Lux		MD	University of Turin/Regina Margherita C	Turin, Italy	data curation, writing - review and editing	
Benedetto	Vitiello		MD	University of Turin/Regina Margherita C	Turin, Italy	conceptualization, supervision, project management, writing - original draft	
Valentina F.	Cudia		MD	University of Turin/Regina Margherita C	Turin, Italy	data curation, writing - review and editing	
Federico	Amianto		MD, PhD	University of Turin/Regina Margherita C	Turin, Italy	data curation, writing - review and editing	
Marta	Borgogno		MD	University of Turin/Regina Margherita C	Turin, Italy	data curation, writing - review and editing	
Federica S.	Ricci		MD	University of Turin/Regina Margherita C	Turin, Italy	data curation, writing - review and editing	
Giulia	Defilippi		MD	University of Turin/Regina Margherita C	Turin, Italy	data curation, writing - review and editing	
Elena	Lonardelli		MD	University of Turin/Regina Margherita C	Turin, Italy	data curation, writing - review and editing	
Claudia	Bondone		MD	Città della Salute e della Scienza	Turin, Italy	data curation, writing - review and editing	
**University of Trieste**							
Giuseppe	Abbracciavento		MD	University of Trieste, B. Garofalo Hospit	Trieste, Italy	data curation, writing - review and editing	
Giorgio	Cozzi		MD, PhD	University of Trieste, B. Garofalo Hospit	Trieste, Italy	data curation, writing - review and editing	
Caterina	Zanus		MD	University of Trieste, B. Garofalo Hospit	Trieste, Italy	data curation, writing - review and editing	
**University of Brescia**							
Thomas	Anfosso		MD	University of Brescia	Brescia, Italy	data curation, writing - review and editing	
Elisa	Fazzi		MD, PhD	University of Brescia	Brescia, Italy	data curation, writing - review and editing	
**Bambino Gesù Children's Hospital**							
Massimo	Apicella		MD	Bambino Gesù Children's Hospital IRCCS	Rome, Italy	data curation, writing - review and editing	
Roberto	Averna		MD, PhD	Bambino Gesù Children's Hospital IRCCS	Rome, Italy	data curation, writing - review and editing	
Umberto	Raucci		MD, PhD	Bambino Gesù Children's Hospital IRCCS	Rome, Italy	data curation, writing - review and editing	
Stefano	Vicari		MD	Bambino Gesù Children's Hospital IRCCS	Rome, Italy	data curation, writing - review and editing	
**University of Cagliari**							
Marzia	Bazzoni		MD	Dept. Biomedical Sciences, Sect. Neuros	Cagliari, Italy	data curation, writing - review and editing	
Sara	Carucci		MD, PhD	Dept. Biomedical Sciences, Sect. Neuros	Cagliari, Italy	data curation, writing - review and editing	
Chiara	Narducci		MD	Dept. Biomedical Sciences, Sect. Neuros	Cagliari, Italy	data curation, writing - review and editing	
Alessandro	Zuddas		MD	Dept. Biomedical Sciences, Sect. Neuros	Cagliari, Italy	conceptualization	

Supplemental Online Content: Nonauthor Collaborators
*First name, last name, and suffix (if applicable) are required and will appear in PubMed.

***First Name and Middle Initial(s)**	***Last Name**	***Suffix (eg, Jr, III)**	Academic Degrees	Institution	Location (city, state/province, country)	Role or Contribution, eg, chair, principal investigator	Group (if more than 1 Group listed in the byline) and/or Subgroup (eg, Steering Committee)					
**Sapienza University**												
Dario	Calderoni		MD	Sapienza University	Rome, Italy	data curation, writing - review and editing						
Luca	Cammisa		MD	Sapienza University	Rome, Italy	data curation, writing - review and editing						
Federica	di Santo		MD	Sapienza University	Rome, Italy	data curation, writing - review and editing						
Arianna	Terrinoni		MD	Sapienza University	Rome, Italy	data curation, writing - review and editing						
Mauro	Ferrara		MD	Sapienza University	Rome, Italy	conceptualization, writing - review and editing						
**University Hospital of Sassari**												
Alessandra	Carta		MD, PhD	Complex Operating Unit of Child Neurop	Sassari, Italy	data curation, writing - review and editing						
Ilaria	Onida		MD	Complex Operating Unit of Child Neurop	Sassari, Italy	data curation, writing - review and editing						
Stefano	Sotgiu		MD, PhD	Complex Operating Unit of Child Neurop	Sassari, Italy	data curation, writing - review and editing						
**IRCCS Istituto G. Gaslini**												
Lino	Nobili		MD, PhD	IRCCS Istituto G. Gaslini/ University of	Genoa, Italy	data curation, writing - review and editing						
Laura	Siri		MD	IRCCS Istituto G. Gaslini, University of G	Genoa, Italy	data curation, writing - review and editing						
Sara	Uccella		MD, PhD	IRCCS Istituto G. Gaslini, University of G	Genoa, Italy	data curation, writing - review and editing						
**Meyer Children's Hospital IRCCS**												
Tiziana	Pisano		MD	Neuroscience Department, Meyer Child	Florence, Italy	data curation, writing - review and editing						
Idanna	Sforzi		MD	Emergency Department and Trauma Ce	Florence, Italy	data curation, writing - review and editing						
Simone	Tavano		MD	Neuroscience Department, Meyer Child	Florence, Italy	data curation, writing - review and editing						
