# Supplementary material for: COVID-19 Pandemic School Disruptions and Acute Mental Health in Children and Adolescents
Source: JAMA Netw Open. 2024 Aug 5;7(8):e2425829. doi: 10.1001/jamanetworkopen.2024.25829 (PMC11301547; doi:10.1001/jamanetworkopen.2024.25829)
Supplement: Supplement 3. — Data Sharing Statement [file jamanetwopen-e2425829-s003.pdf]

Data Sharing Statement

Davico. COVID-19 Pandemic School Disruptions and Acute Mental Health in Children and Adolescents. *JAMA Netw Open*. Published August 05, 2024.
doi:10.1001/jamanetworkopen.2024.25829

Data
Data available: No

Additional Information
Explanation for why data not available: This study was evaluated by the Italian Garante per la Privacy (Italian Data Protection Authority) which has limited the possibility to share the data collected for this study due to privacy concerns.
